# A Qualitative Exploration of Sexual Risk and HIV Testing Behaviors among Men Who Have Sex with Men in Beirut, Lebanon

**DOI:** 10.1371/journal.pone.0045566

**Published:** 2012-09-18

**Authors:** Glenn J. Wagner, Frances M. Aunon, Rachel L. Kaplan, Yashodhara Rana, Danielle Khouri, Johnny Tohme, Jacques Mokhbat

**Affiliations:** 1 RAND Corporation, Santa Monica, California, United States of America; 2 University of California, Berkeley, School of Social Welfare, Mack Center on Mental Health & Social Conflict, Berkeley, California, United States of America; 3 Lebanese AIDS Society, Beirut, Lebanon; 4 Marsa, Sexual Health Centre, Beirut, Lebanon; 5 Division of Infectious Diseases, University Medical Center Rizk Hospital, Beirut, Lebanon; University of Washington, United States of America

## Abstract

Men who have sex with men (MSM) may account for most new HIV infections in Lebanon, yet little is known about the factors that influence sexual risk behavior and HIV testing in this population. Qualitative interviews were conducted with 31 MSM living in Beirut, and content analysis was used to identify emergent themes. Mean age of the participants was 28.4 years, and all identified as either gay (77%) or bisexual (23%). Half reported not using condoms consistently and one quarter had not been HIV-tested. Many described not using condoms with a regular partner in the context of a meaningful relationship, mutual HIV testing, and a desire to not use condoms, suggesting that trust, commitment and intimacy play a role in condom use decisions. Condoms were more likely to be used with casual partners, partners believed to be HIV-positive, and with partners met online where men found it easier to candidly discuss HIV risk. Fear of infection motivated many to get HIV tested and use condoms, but such affect also led some to avoid HIV testing in fear of disease and social stigma if found to be infected. Respondents who were very comfortable with their sexual orientation and who had disclosed their sexuality to family and parents tended to be more likely to use condoms consistently and be tested for HIV. These findings indicate that similar factors influence the condom use and HIV testing of MSM in Beirut as those observed in studies elsewhere of MSM; hence, prevention efforts in Lebanon can likely benefit from lessons learned and interventions developed in other regions, particularly for younger, gay-identified men. Further research is needed to determine how prevention efforts may need to be tailored to address the needs of men who are less integrated into or do not identify with the gay community.

## Introduction

Over half a million people are HIV-infected in the Middle East and North Africa (MENA) region, including over 3,500 in Lebanon [1] where evidence suggests that men who have sex with men (MSM) may account for most new infections [2], [3]. HIV prevalence among MSM in Lebanon is estimated at 3.6% [4], and higher rates of 5–10% are estimated among MSM in other parts of MENA such as Egypt and Sudan [5], [6].

A recent review of the literature revealed several studies of HIV risk behavior had been conducted with MSM in MENA, many of which were conducted in Egypt or Pakistan [3], but few had been implemented in Lebanon. Challenges to conducting such investigations in much of the region include the social and religious taboos that prevent open discussion of sex [7], and because homosexuality is highly stigmatized and even illegal in most countries. The studies that have been done suggest high rates of unprotected anal sex, multiple sex partners, and concurrent sexual relations with women [2], [8], [9]. Studies in Lebanon have revealed that approximately 60% of MSM do not consistently use condoms [10], only 25.0% reported using a condom in their last sexual intercourse [11], and average nearly 10 partners over a year [2], [9]. HIV testing can be instrumental to increasing condom use, particularly among those who are infected [12]; yet the testing rate is thought to be as low as 24% in Lebanon [4]. HIV testing is stigmatized in MENA as it connotes fear of infection and having engaged in inappropriate behavior that warrants the punishment of HIV [13]; for MSM, additional barriers to HIV testing include traditional masculinity and not seeking health services, and internalized homophobia [14].

Research to date has focused mostly on assessing the prevalence of sexual risk behavior and HIV testing, rather than attempts to understand the factors that may influence these behaviors. Drawing on the minority stress model [15], and studies of HIV risk behavior among MSM in other regions, aspects of sexual identity development including self-acceptance and disclosure of one's sexual orientation [16], [17], social influences including sexual stigma, discrimination, and social support [18], as well as relational characteristics such as partner type [19], [20], and trust and commitment within a relationship [21], [22], may influence motivation, decision making and behavior regarding consistent condom use, HIV testing, and other protective health behaviors.

In this paper we report findings from the initial phase of a mixed methods study in which we used qualitative, semi-structured interviews to explore aspects of sexual identity development, sexual risk behavior and HIV testing among MSM in Beirut, Lebanon. Improving our understanding of the psychosocial influences on HIV risk and testing behaviors will help to inform prevention interventions for this much under-studied population.

## Methods

### Ethics Statement

Participants provided oral informed consent prior to being interviewed. Oral, rather than written, informed consent was used because there was no need to collect identifying information and thus the participant's signature on a consent form would have constituted the primary source of risk of loss of confidentiality. The study protocol, including oral consent, was reviewed and approved by the Institutional Review Boards at the RAND Corporation and the Lebanese American University.

### Sample

In Fall 2011, we interviewed 31 MSM living in Beirut as part of a mixed-methods study to explore psychosocial processes that influence sexual risk behavior and HIV testing. The aim of this initial qualitative phase was to inform the methods and measures of the subsequent quantitative phase that will employ respondent driven sampling. Participants were eligible if they were male, age 18 years or older, and reported engaging in any sexual activity with another male in the past year. While any form of sexual activity (oral, anal or mutual masturbation) with a man was sufficient to be eligible, in fact, all participants reported having engaged in anal sex with other men. We purposively sampled younger men such that half (n = 16) were aged 18–25 years, and the others (n = 15) above age 25. Participants were referred by members of our community advisory board and collaborating non-governmental organizations that provide services to sexual minorities; some participants also referred their friends. Participants were paid $30 USD for completing the interview.

### Instrument

Using a semi-structured interview guide, we explored the influence of various social factors on the sexuality and HIV risk behaviour of the respondents. The interviews were used to elicit themes and to determine how common or salient these themes were among an array of respondents. After asking respondents some basic demographic and background related questions, the interview covered the following topic areas: 1) comfort with and disclosure of sexual orientation, 2) experiences of support and/or stigma in response to disclosure, 3) sexual behavior including condom use, 4) HIV testing, and 5) discussion of HIV status, risk and condom use with sex partners.

Interviews were digitally recorded and conducted in the language preferred by the participant (Arabic, French or English). We consulted the community advisory committee to identify a team of five interviewers with experience in qualitative methods who were either MSM or women with a high level of familiarity and comfort with MSM. Participants were allowed to choose the interviewer type.

### Analysis

Interview audio-recordings were translated into English and transcribed verbatim. We utilized a staged technique [23] to identify themes. Two authors (FA and YR) used text management software (*ATLAS.ti*) to mark contiguous blocks of transcript text that pertained to the major themes and subthemes. Three interviews were coded by both FA and YR to ensure consistent application of codes. If there was any disagreement, the coders discussed to reach consensus. Results were aggregated to identify common themes across respondents by characteristics such as consistent condom use and any history of being HIV-tested.

## Results

### Sample Description

Sample characteristics of the 31 men who participated in the study are included in Table 1. Mean age was 28.4 years (SD = 10.5; range: 19–65 years), 87% had at least some college education, and most participants were either employed (55%) or attending university (35%). Among the 27 with known religious affiliation, 52% were Christian and 30% were Muslim.

**Table 1:** Sample Characteristics (N = 31).

Characteristic	N (%)
**Age 18**–**25 years**	16 (51.6)
Mean Age ± SD	28.4±10.5
**At least some college education**	27 (87.1)
**Employment status**	
Employed	17 (54.8)
In school	11 (35.5)
Unemployed	3 (9.7)
**Religious affiliation**	
Muslim	8 (25.8)
Christian	14 (45.2)
No affiliation	5 (16.1)
No response	4 (12.9)
**In a relationship currently**	9 (29.0)
**Sexual orientation**	
Homosexual	24 (77.4)
Bisexual	7 (22.6)
**Comfort with sexual orientation**	
Very comfortable	11 (35.5)
Some level of discomfort	20 (64.5)
**Has disclosed to any family members**	24 (77.4)
**Has disclosed to a parent**	15 (48.4)
**Family response to disclosure (if disclosed to)**	
Highly stigmatizing	4 (16.7)
Received at least some support	20 (83.3)
**Mean (SD) number of sex partner per year**	25.3±35.2
**Consistent condom use**	16 (51.6)
**Discusses HIV risk with sex partner(s)**	
Mostly	8 (25.8)
Sometimes	13 (41.9)
Never	10 (32.3)
**Has tested for HIV**	24 (77.4)

### Processes of Sexual Identity Development

#### Comfort with sexual orientation

Each participant self-identified as being either gay (n = 24; 77%) or bisexual (n = 7). Eleven men reported feeling very comfortable with their sexual orientation, with statements such as, *“[I am] very comfortable [about being homosexual] because I know that it is not a choice. I know that it is not something I can change. I am not sad about it.”* Another 14 men also expressed some comfort but there remained suggestions of internal struggles, often as a product of perceived stigma from others: *“I am very comfortable [with my sexual orientation], but because I can't do it in public…Everything that you want to do you have to in secret, it is not open. That is what hurts.”* Another respondent described how the workplace is an area of his life that highlights some remaining discomfort, *“I am on the better side of comfortable. It is always a process, so what happened is the circle of my comfort is getting bigger as I live. Professionally I am still uncomfortable. I want to separate my sexual identity from what I do [work-wise], especially in Lebanon because I don't think it fits.”*

Six men reported significant discomfort with their sexual orientation. Religious beliefs that homosexuality is immoral and a desire to remain true to one's faith, as well as unsuccessful attempts to control desires for sex with men, were prominent in the descriptions of this discomfort, *“I feel guilty a lot of times since I do believe that prophet Mohamed prohibited sex between men and every time I pray, I really feel guilty.”* Another respondent gave the following statement:

*“I am not comfortable and at times I wanted to change it, but I came to realize that I can't. Now I try not to make it a lifestyle, but I still need to sleep with men from time to time. There are a lot of reasons [I am not comfortable]. First, there's the religious part. I am a believer and I know that in the bible homosexuality is an abomination. Besides, I know that in order to be successful in this society I have to get married and have kids, and my homosexuality prevents me from doing that, therefore I hate it.”*

#### Disclosure of sexual orientation

All participants reported that someone in their social network knew that they had sex with men, and nearly all said that some or most of their friends knew, though they were often other MSM. However, while three-quarters of the sample indicated that someone in their family knew that they had sex with men, only half (n = 15) reported that at least one parent (usually the mother) was aware. A third of participants indicated that co-workers or fellow students knew of their sexual orientation. Comfort level with one's sexual orientation seemed to be related to the level of disclosure or being “out”; 8 of 15 men who had disclosed to parents described being very comfortable with their sexual orientation compared to just 3 of the 16 who had not told a parent.

For some, the process of others becoming aware of the participant's sexual orientation involved direct disclosure (where the participant told the third party), but for many disclosure was more indirect, where the participant did not directly disclose their sexuality openly but nevertheless assumed someone suspected their sexual orientation. One participant described how he feels uncomfortable directly telling his parents, but does not deny, which could suggest a desire for his parents to know on some level: *“My mom has a lot of doubts. Once, I had something on my neck. My mum asked me what this is. I told her, ‘I don't know. This is from the party I had.' She replied, ‘Is it a man or a woman?’ I said, ‘I don’t know’”; “I never hide it. I never discussed it with my parents but I know that they know.”*

#### Receipt of support versus stigma in response to disclosure

Two-thirds of the sample described the responses of others who became aware of their sexual orientation as being mostly supportive. Forms of support ranged from emotional support and encouragement, to simply being accepting and not treated any differently.

*“I got the support of my family. They said that they love me no matter what. My dad also said that he used to suspect one of his cousins, and when this last one came out, he was the only one who supported him, so that was very relieving. My sister is my biggest supporter. We always joke about who's going to get this guy or that guy.”*

Some recipients of the disclosure demonstrated immediate support, but others had a difficult time at first before eventually becoming supportive. A few participants received a mix of supportive and non-supportive responses, as exemplified in the following quote:

*“The friend I told back in school didn't react in a bad way at first. He wasn't shocked or anything, but when he went around and told people, they started making fun of me in ways that didn't happen before. Two people, who were already friends with me, became even better friends when they found out. Then my brother found out. He was normal; he wasn't supportive and he wasn't unsupportive. We have a good relationship, but we don't talk about personal things a lot.”*

Four men reported strong ridicule or lack of acceptance from others, and these experiences involved being rejected by family and needing to leave home. One respondent described being afraid of physical violence if his family found out, *“If they know I am homosexual, especially my brother, [they] will kill me. If I knew he found out, I'd escape.”* Another respondent described an ongoing emotional torment that resulted in him leaving home and his community:

*“I have been treated badly since I was a teenager at school. They used to make fun of me since I used to spend my time with girls more than boys…This has hurt me a lot and created a lot conflicts inside my family. That's why I left the house and the street and moved to another place. It was very hard, and I used to stay alone because I barely had friends. I felt different from the start.”*

### Sexual and HIV Testing Behavior

#### Sexual history and partners

The average age of initiation of sex (which was typically mutual masturbation) among the study participants was 13.9 years (range: 9 to 24 years), and roughly half of the participants had their first sexual encounter with another man. The median reported number of male sex partners over the past year was 8, while the mean was 25.3 and the range was from 1 to 130. None of the men reported having sex in exchange for money or gifts. Ten respondents reported recent sexual activity with both men and women. Nine respondents reported being in a current committed relationship with another man, and two were currently married to a woman. When asked to describe their sexual activity with men, nearly all men reported engaging in both oral and anal sex with men, and most engaged in both receptive and insertive anal sex.

#### Perception of “risky” sexual behavior and condom use

When asked to describe what they consider to be “risky” sexual behavior, nearly all participants cited anal sex without the use of condoms. Some participants described condoms as being more important during receptive anal sex, but most conceded that condoms were important for risk reduction with insertive anal sex as well. A few men added that swallowing semen was risky, and one respondent included giving oral sex with sores or cuts in one's mouth, but most participants felt that oral sex without condoms was not risky, or at most minimally risky. None reported using condoms during oral sex. Other behaviors or sexual contexts that were named as risky included having sex with risky partners [e.g., participants named drug users, sex workers, promiscuous or anonymous partners, partners with sexually transmitted infections (STIs)], sharing intravenous needles, using substances in the context of sex, and fisting–reflecting an accurate knowledge of HIV risk.

With regard to anal sex, 15 reported inconsistent condom use, while 11 reported always using condoms. The remaining 5 participants were in committed relationships and stated that they always use condoms but not with their primary partner; some of these men stated that their relationship was monogamous and that mutual HIV testing with the partner was done in order to justify not using condoms during sex with each other, but this information was not systematically elicited. Nonetheless, for the purposes of making comparisons between those who engage in safe and unsafe anal sex, we merged these 5 men with the 11 men who always use condoms in order to comprise the subgroup who have “safer” sex.

#### HIV status and history of HIV testing

Twenty men reported receiving a negative result at their last HIV test, 1 participant reported being HIV positive, 3 men were tested but did not share their results, and the remaining 7 men had never been tested. Nearly half (n = 11) of the 24 participants who had tested for HIV reported testing on a regular basis, ranging from every two months to once per year. Two thirds (n = 16) of the 24 men who had been tested were using condoms consistently compared to none of the 7 men who had never been tested.

### Factors influencing condom use and HIV testing

#### Characteristics of the sexual partner and relationship

Decisions and behaviors related to condom use and HIV testing often centered around the nature of the sexual partnership and relationship dynamics. While several men (n = 10) stated that they never discuss HIV status and risk with sex partners, most respondents reported discussing HIV with some but not all partners. Several partner characteristics influenced the decision of whether to discuss HIV risk, as described below, and circumstances related to condom use and HIV testing were often raised in this context.

#### Regular versus casual partners

Several men indicated that they do not discuss HIV with casual partners, but they do once some sort of relationship or regularity in sexual relations develops with the partner, as revealed in this response, *“[I talk with my partners about HIV] when I get the feeling that it's starting to go beyond dating into something more serious.”*

Over half of the sample (n = 17) reported that they did not use condoms during sex with partners with whom they were either in a relationship or who were regular sex partners. The desire to have unprotected sex within the context of a relationship and with one's primary partner seemed to be a key motivator of discussions of HIV risk and also HIV testing as revealed in this response, *“My boyfriend and I got tested together, because if it is serious and we want to have sex without a condom obviously we have to get tested.”* Similarly, another respondent stated, *“At the beginning of the relationship, when [my boyfriend and I] started having sex, we used condom. We discussed the necessity to get tested so we can remove the condom.”*

For other men, discussing HIV status and risk was prominent in negotiations with potential casual partners, particularly in the context of meeting sex partners online. The relative anonymity of the internet made it easier for some men to initiate the discussion of HIV status and condom use, and to be more forthright, candid and less concerned about rejection. A respondent who uses MSM-oriented social networking websites to meet partners explained how this forum provides a unique opportunity to discuss sensitive topics prior to meeting in person, *“Since most of my meetings are through the internet, [discussing HIV/STI status] happens before meeting while we are chatting [online]. When you are meeting someone in a club, usually you don't have a lot of time to discuss such issues.”* While these websites provide a more comfortable environment for discussing such personal matters, he also cautioned that the information might not always be accurate, *“When it comes to the internet, what you are told is not always right, it is not always accurate, a lot of people might hide a lot of things. After all it is the internet; you can say whatever you want.”*

#### Male versus female partners

Some of the respondents who also have sex with women indicated that while they discuss HIV with male partners, they do not with female partners. One respondent described how he associates HIV risk and the need to discuss HIV only with men, as HIV is thought to be more of a “gay thing”, even though he is aware that women also contract HIV. *“If [my partner] was a woman, I might not even ask her [about her HIV status], it wouldn't even come up. I usually have this predisposition of thinking that it is just the gays [that have HIV], but of course it is not*.*”* Other men were reluctant to discuss HIV with their female partners in fear that it might lead the woman to suspect that the respondent has sex with men or may lead to her rejecting sex. However, while men often did not discuss HIV risk with their female partner, a number of respondents did indicate that condoms were used with these women, including sometimes at the insistence of the woman, because of a desire to prevent pregnancy. One respondent stated, *“In Beirut, most girls require the use of condoms because they are scared of getting pregnant, and they are scared of scandal.”*

#### Perceived HIV status of partner

Some participants described opting to use condoms if they suspected a partner might have HIV instead of initiating the potential uncomfortable conversation of HIV status, *“We never open the subject, but once I met a man with buttons [spots] all over his body. When I see something I don't like I use a condom right away. It is not easy to open the subject and in any case they will not tell the truth*.*”* Serosorting, or having sex only with men of the same HIV status, was not specifically mentioned by respondents, but was implied in some responses, *“[If someone hasn't been tested], I get scared and I stop. It has happened to me twice and after I realized that the condom broke I said I can't do it anymore because I get scared*.*”* Another person said, *“The people I sleep with, I would know who they are, what they are like. I wouldn't sleep with someone I hadn't seen before*.*”*

#### Anxiety and fear related to infection and disease

Several of the men who use condoms consistently spoke of fear of infections and the dangers of HIV and other STIs when explaining why they engage in safer sex. For example, one respondent said, *“No, I am very safe. I am very scared for myself*,*”* and others even spoke of condom use in life and death terms: *“Risky sex is a way to commit suicide without minimal protection,”* and *“Lots of gays have HIV so I am not taking the risk to end my life just for sex.”* One participant talked about rarely engaging in anal sex because of the fear of risk of sexually transmitted infections (STIs) because, *“the fear is greater than the pleasure I get out of it… for me, I only do it with someone I know and is safe, or with someone I am in a relationship with. But every time I do it, once every 2 years, I am always safe when I do it, (and use) extra precautions.”*

While fear and anxiety about HIV infection undoubtedly contributed to some men getting tested for HIV, negative affect was also associated with decisions not to be tested. Most of the men who had an HIV test described a “professional” and comfortable testing experience. *“[HIV testing] was good. They did the test and tell me what to avoid*.*”* Any discomfort was usually associated with the anxiety of testing, and not the testing facilities' lack of professionalism.

However, the 7 participants who reported never having had an HIV test expressed concerns about the confidentiality of the HIV test results and the implications of a potential positive result both in terms of the medical disease and the stigmatizing response from others. One respondent explained his concerns as follows, *“I might be afraid to reveal my name or even afraid from the result knowing I have been unsafe a couple of times*.*”* Another respondent shared, *“It's a scary disease, there's no cure. Besides, if society finds out that you have AIDS, they will exclude you.”*

Fear and anxiety are also barriers to discussing HIV risk and condom use with sex partners, although this affect was not related to fear of infection but relational dynamics such as rejection. Responses about the barriers to discussing HIV status and risk highlighted an emotional component to the decision process. Respondents commonly spoke of such discussions as being awkward and uncomfortable, especially with regard to initiating the discussion as many said they have no problem discussing HIV if the other person raises the issue. Others spoke of avoiding the discussion, at least until after having sex, because the subject of HIV “ruins the moment” or is “scary…. freaks me out,” and thus threatens the likelihood of the sexual encounter being a pleasurable experience. Similarly, some were reluctant to discuss HIV for fear that the partner would be turned off or insulted, or result in loss of an opportunity to have sex, *“It is usually not a topic to talk about when you are in bed. If you want to turn the person on, you don't talk about it.”* Some feared that discussing HIV status would lead their sex partner to assume they are HIV positive and to rejection, *“I tried [talking about HIV status] once and then the guy I was chatting with thought I was talking about it because I am positive. So I've never done it again*.*”*

#### Sexual identity development and sexual risk behavior

The respondents did not speak directly about their condom use or HIV testing being associated with their comfort with and disclosure of their sexual orientation. Individuals may be less consciously aware of the connections between sexual risk behaviors and processes of sexual identity development compared to other factors influencing such behavior, but we sought to explore these associations in our data in order to inform the quantitative assessments in the next phase of our research.

Our data suggest that processes of sexual identity development, namely comfort level with one's sexual orientation and the level of MSM disclosure to parents and family, may play a role in sexual risk behavior and HIV testing. Just under half (7/16) of the safer sex group stated they were very comfortable with their sexual orientation compared to a quarter (4/15) of the unsafe men, and nearly two-thirds of the safer sex group (10 of 16) had disclosed their MSM status to a parent, compared to a third (5/15) of the unsafe sex group. Furthermore, two-thirds (n = 9) of the 13 participants who were both uncomfortable with their sexual orientation and had not disclosed to either parent reported inconsistent condom use compared to just one-third (n = 6) of the other 18 men in the sample.

Similar to our findings related to condom use, many of the men (10 of 24) who had been tested for HIV stated they were very comfortable with their sexual orientation compared to just one of the 7 untested men. Among the HIV-tested men, over half (13/24) had a parent who knew they had sex with men, compared to only one of the men who had not tested.

## Discussion

This is one of the first published qualitative studies to explore the psychosocial processes of sexual identity development and HIV sexual risk behaviors among MSM in Lebanon and the larger MENA region. Our findings suggest that much like studies of MSM in other areas of the world [24], decisions and behaviors regarding condom use and HIV testing among MSM in Beirut are influenced by a wide array of individual, interpersonal and social factors including fears and anxiety about disease infection [25], relationship factors including trust, intimacy and commitment [21], [22], and processes of social identity development [16], [17].

Relationship context or partner type was perhaps the strongest factor influencing the use of condoms during anal sex. Many participants spoke of always using condoms with casual partners, but that once a meaningful relationship began with someone, condom use was often discontinued. In this context, discontinuation of condom use seemed to reflect a milestone in the relationship, and one that connoted trust and commitment. The role of trust, commitment and intimacy in the decisions of male couples to not use condoms has been well documented [21], [22]. Unprotected sex in the context of a committed relationship can be a rational choice, particularly when both partners have tested HIV-seronegative and are monogamous, both of which were noted by some of the men in our study. However, non-monogamy is fairly normative in gay relationships [26], [27], including in Lebanon, and research indicates that HIV transmission most often occurs in the context of sex with a regular, primary partner [28], all of which suggests that committed relationships are not necessarily a safe haven against HIV and other sexually transmitted infections. HIV prevention interventions that target men in relationships need to account for the needs of couples to demonstrate love, trust and commitment to each other and how these needs and feelings can be incorporated into skill development for effective communication about and negotiation for consistent condom use.

Some of these partnership dynamics extend beyond sex in the context of committed relationships. Some men described not using condoms with regular partners or men they knew well, and with whom they were not in a romantic relationship. In this context, men described knowing the partner well enough to be confident that they are healthy (i.e., HIV and STI free), and able to trust that the partner would inform them of any risk of transmission if the partner had an STI, and thus are comfortable with not using condoms. These assumptions have been shown in prior research to often not be accurate and to present opportunities for exposure to risk of infection [29]. Hence, the need for prevention interventions to highlight how the need for condoms remains even with regular, well-known partners, and that much like the strategies for couples mentioned above, values of trust, caring for one another and the need for condoms can be presented as complimentary and not discrepant.

Consistent with men reporting more vigilant condom use with casual partners, our respondents also described being more comfortable initiating discussions about HIV status, risk and condom use with men they meet online in gay social networking websites. The use of internet social networking sites and mobile phone applications for meeting sex partners is common among MSM [30], including in Lebanon. The relative anonymity provided by online communications may be especially attractive as an outlook for sexual expression in cultural contexts such as MENA where homosexuality is highly stigmatized; yet, while men may feel more comfortable initiating discussions about HIV status and condom use in this context [31], some studies reveal that MSM who use the internet to meet sex partners tend to engage in higher risk sexual behaviors [32], [33], although other studies have not found such an association [31]. Research on sexual risk behavior and negotiation in the context of online connections among MSM in MENA is needed to better understand how HIV prevention interventions for online users may need to be culturally adapted for MENA.

Emotional states such as fear and anxiety emerged as playing key roles in both condom use and HIV testing. Anxiety related to fear of infection motivated many participants to always use condoms, lending credence to prevention intervention messages that emphasize the negative consequences of unsafe sex as opposed to the health benefits of safe sex [34], although the motivation to remain healthy may be inherent in fears about infection. Fear of infection can also motivate HIV testing, but our respondents revealed how anxiety and fear can often impede HIV testing as men are afraid of learning that they are infected with a disease that cannot be cured and the treatment of which may be hard to obtain. Access to affordable HIV treatment is thought to be associated with increased HIV testing rates [35]; in Lebanon, HIV antiretroviral medications and HIV testing can be obtained for free, but not HIV care more generally and many do not have health insurance. Avoidance of HIV testing was also associated with the fear of social stigma that might follow receipt of an HIV diagnosis, highlighting the role of stigma towards people living with HIV, as well as homosexuality, as impediments to HIV prevention and testing.

Our data also highlight the potential role of sexual identity development in sexual risk behavior and HIV prevention. Although causality cannot be inferred from our cross-sectional data, and the small, convenience sample not withstanding, it is noteworthy that respondents who expressed being very comfortable with their sexual orientation and who had disclosed their sexuality to family and parents tended to be more likely to use condoms consistently and to be tested for HIV. Other studies with MSM have shown a relationship between self-acceptance and disclosure of sexual orientation and lower levels of sexual risk behavior [16]. We hypothesize that comfort with and disclosure of sexual orientation and same-sex sexual activity may influence sexual health and risk behaviors through their relationship to self-esteem, motivation and confidence to engage in healthy protective behaviors, and facilitation of access to prevention information. We will be able to explore these relationships more systematically and reliably in the quantitative phase of our research with this population, which is currently ongoing. If future research corroborates the value of comfort with and disclosure of sexual orientation as processes that promote healthy sexual behavior, then such findings would suggest the value of interventions that facilitate effective disclosure decision making and the acknowledgment of the rights of sexual minorities. However, such intervention strategies would need to account for not only the potential benefits, but also the risks (e.g., violence, rejection, discrimination) associated with disclosure of same-sex sexual activity and advocacy of sexual minority rights, given the conservative and highly stigmatizing traditional values of Lebanon and the larger MENA region regarding homosexuality.

The primary limitation of this study lies in our findings not being transferable to the larger MSM population in Lebanon. The sample was comprised of mostly young, well-educated and gay-identified men; hence, our findings do not necessarily represent the experience of MSM who are older, closeted, heterosexually-identified, poorly educated or living in the more conservative areas outside of greater Beirut. We had attempted to recruit heterosexually-identified MSM, but this proved more difficult than anticipated, although nearly a third of the participants did report having recent sex with women. Innovative strategies will be needed to overcome the challenges of engaging men who are less integrated into the gay community or not identifying as gay or bisexual, as these men are likely to be more isolated, have less access to support and information, and thus be more vulnerable to engaging in risky behaviors. We have recently begun the next phase of this study which employs respondent driven sampling; it is our hope that this methodology will enable us to penetrate more segments of the MSM community, and thus enable a more robust examination of the patterns and relationships between constructs that we have observed in the qualitative data.

In summary, our findings indicate that the factors influencing the HIV sexual risk and testing behaviors of MSM in Beirut are similar to those found in studies of MSM in other parts of the world, namely relationship and partner characteristics and dynamics, fear and anxiety related to disease infection and stigmatization, and social and psychological processes in the development of sexual identity. This suggests that prevention efforts targeting MSM in Lebanon can benefit from lessons learned and interventions developed and tested in other regions, particularly for younger men who self-identify as gay or bisexual. However, for men who are less integrated into the gay community or do not self-identify as gay or bisexual, which may be a substantial part of the MSM population in Lebanon and other parts of MENA, further research is needed to determine how prevention efforts may need to be tailored to address their needs, and whether the predominant local culture presents unique challenges that are yet to be identified.
